# A phylogenetic contribution to understanding the panzootic spread of African swine fever: from the global to the local scale

**DOI:** 10.1093/ve/veaf103

**Published:** 2025-12-24

**Authors:** Gianluigi Rossi, E Carol McWilliam Leitch, Jake Graham, Roberta Biccheri, Carmen Iscaro, Claudia Torresi, Samantha J Lycett, Francesco Feliziani, Monica Giammarioli

**Affiliations:** Centre of Expertise on Animal Disease Outbreak (EPIC), Scotland; The Roslin Institute, R(D)SVS, University of Edinburgh, Easter Bush Campus, Midlothian, EH25 9RG, Scotland; The Roslin Institute, R(D)SVS, University of Edinburgh, Easter Bush Campus, Midlothian, EH25 9RG, Scotland; The Roslin Institute, R(D)SVS, University of Edinburgh, Easter Bush Campus, Midlothian, EH25 9RG, Scotland; National Reference Center for Swine Fever (CEREP), Istituto Zooprofilattico Sperimentale Umbria e Marche “Togo Rosati”, Via Gaetano Salvemini, 1, 06126, Perugia, Italy; National Reference Center for Swine Fever (CEREP), Istituto Zooprofilattico Sperimentale Umbria e Marche “Togo Rosati”, Via Gaetano Salvemini, 1, 06126, Perugia, Italy; National Reference Center for Swine Fever (CEREP), Istituto Zooprofilattico Sperimentale Umbria e Marche “Togo Rosati”, Via Gaetano Salvemini, 1, 06126, Perugia, Italy; Centre of Expertise on Animal Disease Outbreak (EPIC), Scotland; The Roslin Institute, R(D)SVS, University of Edinburgh, Easter Bush Campus, Midlothian, EH25 9RG, Scotland; National Reference Center for Swine Fever (CEREP), Istituto Zooprofilattico Sperimentale Umbria e Marche “Togo Rosati”, Via Gaetano Salvemini, 1, 06126, Perugia, Italy; National Reference Center for Swine Fever (CEREP), Istituto Zooprofilattico Sperimentale Umbria e Marche “Togo Rosati”, Via Gaetano Salvemini, 1, 06126, Perugia, Italy

**Keywords:** whole-genome sequences, phylodynamics, molecular epidemiology, DNA virus, suid disease, ASF

## Abstract

African Swine Fever virus has become a primary concern for veterinarian health agencies and pig producers worldwide. The current panzootic of the virus genotype II is having a devastating impact on pig production in Africa, Europe, Asia, Oceania, and Hispaniola (Caribbean). Due to its high persistence and mortality rate, disease control policies require enhanced passive surveillance, wild boar depopulation, containment, and other costly interventions, as a safe and effective vaccine is not currently available. Since 2007, several disease clusters have emerged far from both its original range (South-Eastern Africa) and from other affected suid populations. These transmissions were likely caused by anthropogenic movement, facilitated by the virus persistence in the environment and on contaminated material. The objective of this research was to understand the spatio-temporal dynamics of the African Swine Fever virus panzootic, with a specific focus on clusters from mainland Italy. We mapped and analysed the virus spread using 228 whole-genome sequences available from online repositories and from the Italian cases/outbreaks, combined with their metadata. We inferred pathogen phylogenies using a Bayesian phylodynamic model, with which we obtained a time-scaled and spatially explicit maximum clade credibility tree. Our results indicate that the Eurasian genotype II panzootic originated in Africa around 20 years ago (September 2003–May 2007) and showed long-distance transmissions across regions or continents within a short time frame, including from Europe to East Asia and from South-Eastern to Western Africa. Dense local dynamics, particularly in areas where the disease affected a naïve population, were also observed. The distribution of spatial distances inferred along the trees’ branches further highlighted these trends and revealed how previously observed survival times in pork products could allow the virus to traverse distances up to 900 km (in 137 days). Finally, from the available data, we identified at least seven separate introductions in Europe, of which at least three caused new clusters on mainland Italy. This study provides important insights on the African Swine Fever virus introduction into many affected areas worldwide and highlights the crucial role of genomic surveillance in correctly tracking the pathogen spread and monitoring the virus potential evolution.

## Introduction

African Swine Fever virus (ASFV) is one of the most serious threats to the global pig industry. The current panzootic is causing major economic losses worldwide, including in China (You et al. 2021) and the European Union (Niemi 2020), the two world’s leading pigmeat producers and exporters. In South-East and Pacific Asia, ASFV is also decimating the population of native suids, threatening the food security of many rural communities (Meijaard et al. 2024).

The pathogen is a large (170–193 kbp) double-stranded DNA virus, first identified in Eastern Africa in 1921 (Eustace 1921), where it is maintained through a sylvatic cycle involving warthogs, mostly asymptomatic hosts, and soft ticks of the *Ornithodoros* genus (Dixon et al. 2019). The disease causes a haemorrhagic fever in domestic pigs and wild boars, with virulent strains that can lead to up to 100% mortality in naïve populations (Dixon et al. 2019). Currently, a safe and effective vaccine does not exist (Zhang et al. 2023a); thus, control strategies rely on mechanically preventing transmission (e.g. fencing, biosecurity, control zones) and culling (Sánchez-Vizcaíno et al. 2015, Blome et al. 2020). One concerning characteristic of ASFV is its ability to survive in the environment and in uncooked pigmeat products, such as cured meat (Petrini et al. 2019). Consequently, movement of contaminated material can lead to new clusters in areas outside its original range.

The current ASF panzootic is caused by the genotype II: it was first identified in Georgia and in other Caucasus countries in 2007, including several Russian republics (Costard et al. 2009). From the Trans-Caucasian region, ASFV spread to Eastern Europe, reaching the European Union in the Baltic countries and Poland in 2014, then to Central Europe and to the Balkan region. It further spread as far as Belgium (2018), Italy (2022), Sweden (2023), and Albania (2024) (WAHIS, 2026). In 2018, it emerged in China (Zhou et al. 2018) and between 2018 and 2019 in other East Asian countries, including Mongolia, Vietnam, Cambodia, Hong Kong, North Korea, South Korea, Laos, the Philippines, Myanmar, Indonesia, and Timor Leste (Mighell and Ward 2021, Shao et al. 2022). In 2021, it was detected on the island of Hispaniola, in the Caribbean (Haiti and Dominican Republic) (Jean-Pierre et al. 2022). In many of the affected regions, ASFV has been successfully established in the local wild boar population, which acts as a reservoir for spillover to domestic pigs (Brown et al. 2024).

This global spread is likely caused by multiple transmission routes, which allow the virus to be spread at different spatial scales. Vector-mediated and animal-to-animal transmissions, either between domestic pigs and wild boars or between the two, can spread the disease at the local spatial scale. However, anthropogenic-mediated transmission such as fomites on farm visitors’ clothing, equipment, or vehicles can reach wider areas in a short time span (Mazur-Panasiuk et al. 2019). Finally, ASF-contaminated food, animal feed, or other material can spread the virus at the intercontinental scale (Mur et al. 2012).

In Italy, ASFV genotype II was detected for the first time in a wild boar carcass in early January 2022, in the North-Western province of Alessandria (Piemonte region) (Iscaro et al. 2022). Since then, the cluster has expanded to include another four regions (Liguria, Lombardia, Emilia Romagna, and Toscana), while another three clusters have emerged in noncontiguous regions: one in Lazio, within the Rome municipality (2022), one in Calabria (2023), and finally one in Campania (2023). The Lazio cluster was resolved in January 2025, thanks to a planned and coordinated eradication effort (carcasses active search, containment of the infected population by artificial barriers, wild boar depopulation by low-impact methods only). In October 2025, Calabria was also declared free from ASF (European Union, 2025), whereas the other two are currently ongoing. From previous genome analyses (Giammarioli et al. 2023a, 2023b, 2024), it emerged that the strains sampled in some of these clusters belong to different genetic groups, so questions were raised about the virus introduction pathway in the country.

Here, we aim to untangle the spatio-temporal dynamics of the ASFV genotype II spread by using a phylogenetic modelling approach, which exploits the mutations in the viral genome, coupled with the isolates’ metadata, to obtain georeferenced time-scaled phylogenies. We used an alignment that included 146 whole-genome sequences (WGSs) deposited in GenBank (Sayers et al., 2025), and 82 sequences isolated across the four Italian mainland clusters. We first analysed the global spread of ASF to understand the outbreak detection timeline. We then focused on the four Italian clusters and their potential links with other ASFV strains detected in Europe and Asia.

Our results unveiled the global dispersal dynamic of the virus genotype II and shed light on the estimated transitions between different geographical regions. Finally, we have demonstrated that the Italian clusters are probably the result of multiple introductions of viral strains from elsewhere in Eurasia.

## Material and methods

### Alignment assembling and data preparation

A total of 228 ASF virus genotype II whole virus WGSs were collected for the analyses. We sourced 146 WGSs from the GenBank online repository, representing most of the affected continents with available WGSs up to January 2024 (NCBI). Another 82 were obtained from strains sampled in the four mainland Italian clusters: from the North-West (samples from Piedmont and Liguria, the initially affected regions), Rome municipality, Campania, and Calabria (see Table S1 for the full list of WGSs). The software MAFFT v7.511 (Katoh and Standley 2013) was used to perform a multiple sequence alignment.

**Figure 1:**
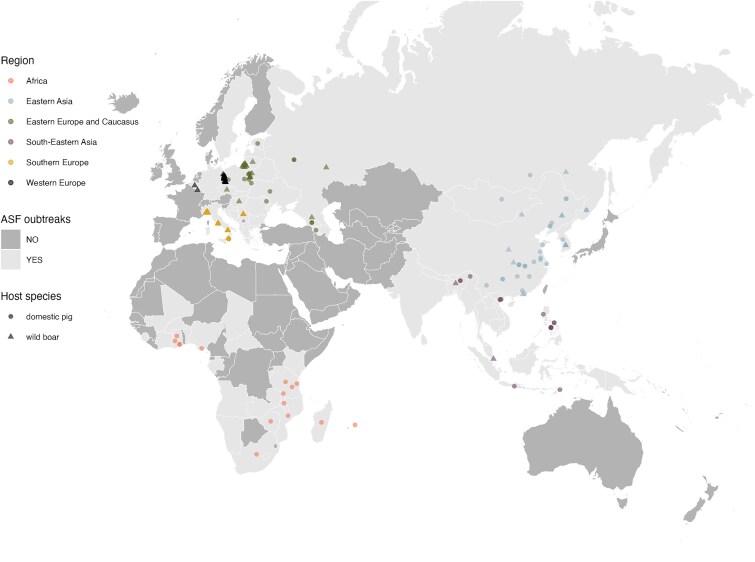
The locations of the 228 African Swine Fever virus (ASFV) whole-genome sequences used in this study. Countries coloured in light grey have recorded ASFV clusters or cases since 2007 (data from Empres-Plus, updated to April 2024). Haiti and the Dominican Republic (Caribbean) are the only countries with reported clusters of ASFV not shown on the map. The sequences’ locations are represented by dots for domestic pigs or by triangles for wild boar (or feral pigs), and they are coloured depending on the assigned region.

To run the analyses of the ASFV WGSs, we used the associated metadata to the best of our knowledge, namely, sample date, host (wild boar or domestic pig), and geographical location. For the latter, we used the available geographic coordinates and a macro-region assigned according to the United Nations Geoscheme (see Supplementary Material for more information). The WGS’s location and region are reported in Fig. 1.

### Time-scaled trees and traits

To obtain a time-scaled phylogeny and the highest clade credibility tree, we used the Bayesian Evolutionary Analysis Sampling Trees (*BEAST*) v1.10.5 (Suchard et al. 2018), with the BEAGLE library (Ayres et al. 2019). The *BEAST* software uses Markov Chain Monte Carlo (MCMC) algorithms to infer values taken by multiple evolutionary parameters, such as divergence times and evolutionary rates, by repeated sampling of probability distributions. A first set of analyses was carried out with the software *IQ-TREE2* (Minh et al. 2020)*,* to identify an initial substitution model candidate and obtain a maximum-likelihood tree. This was, in turn, tested with the software *Tempest v1.5.3* (Rambaut et al. 2016) to assess the presence of enough genetic signal in the sampled viral population. Several preliminary models in *BEAST* were run, to select the best evolutionary model, and compared using Marginal Likelihood Estimation (MLE), calculated with the Path sampling and Stepping-stone sampling methods (Baele et al. 2012) (see Supplementary material). The sampling date of sequences with uncertain date (only year or year and month) was also estimated by the software (Shapiro et al. 2011). Once the best phylogenetic model was identified, the model was run again using alternative chain lengths and random number seeds to improve variability.

The obtained posterior tree samples were used as input for a further run in *BEAST* to generate a new tree distribution with trait partitions: two discrete traits, identifying the isolates region (Table 1) and host (wild or domestic), and one continuous (geographical coordinates). The model used for the phylogeographic reconstruction in continuous space was the Brownian random walk (Lemey et al. 2010, Pybus et al. 2012). The chain length was 10^8^, sampled every 10^4^ to obtain 10 001 trees. We focused our analysis on the final 9001 trees, after discarding 1000 as burn-in. The maximum clade credibility (MCC) tree was extracted with *TreeAnnotator* v1.10.4 and visualized with *FigTree v1.4.4* (FigTree, n.d.), while the results were plotted in *R v4.4.1* (R Core Team 2024).

**Table 1:** Summary of the number of ASF whole-genome sequences included in the study, divided by region and host. Armenia and Estonia had one sequence each, but they were excluded after the quality assessment. See Fig. S1 for a timeline of the sampled sequences.

Region	Host	# total sequences	# selected sequences	Min year	Max year	Represented countries
Africa	Domestic pig	15	15	1998	2022	Ghana, Madagascar, Malawi, Mauritius, Mozambique, Nigeria, South Africa, Tanzania, Zimbabwe
Eastern Asia	Domestic pig	30	25	2018	2022	China, Mongolia, South Korea, East Russia
	Wild boar	14	14	2018	2020	
South-Eastern Asia	Domestic pig	21	21	2019	2023	India, Indonesia, Philippines, Singapore, Timor-Leste, Viet Nam
	Wild boar	2	2	2020	2023	
Eastern Europe and Caucasus	Domestic pig	16	12	2007	2019	Czech Republic, Georgia, Hungary, Lithuania, Moldova, Poland, West Russia, Ukraine (Armenia, Estonia)
	Wild boar	18	18	2014	2019	
Southern Europe	Domestic pig	12	11	2022	2023	Italy, Serbia
	Wild boar	75	74	2019	2023	
Western Europe	Domestic pig	2	2	2021	2021	Belgium, Germany
	Wild boar	23	23	2018	2021	

### Analysis of the dispersal distance along branches

The phylogeographic spatial diffusion model described above allows *BEAST* to estimate the spatial coordinates of all internal nodes in the time-scaled trees, and therefore, we can obtain an estimate of dispersal distances along the tree branches across the posterior set of 9001 trees. However, because the inferred spatial coordinates of internal nodes are in continuous space, these might fall in geographic areas or regions with no disease observation or, in some cases, geographic areas lacking a host species. Consequently, these estimated internal node locations might bias the branches’ dispersal distance estimates, hindering potential analyses on such distance distribution, especially in relation to the branches’ dispersal time.

Therefore, we simplified the internal node structure of parts of the trees; we recalculated branch distances as going from (grand)parent internal nodes located in ASFV-affected countries to nodes (or tips) in ASFV-affected countries, bypassing the nodes located in presumed ASFV-free countries. The ASFV status of each country was defined from the case data published on FAO’s Empress-I (Empres-Plus, n.d.). The dispersal distances were also assessed according to their dispersal time, i.e. the time between the accepted parent node and the node (or tip) estimated or observed time. We analysed the dispersal distance distribution according to a set of time difference thresholds, chosen according to the virus persistence in different substrates: up to 15 days, approximately corresponding to the half-life of ASFV under shipping conditions [14.2 days in (Stoian et al. 2019)], 102 days (15 weeks), corresponding to the lifespan of the virus in refrigerated meat (Blome et al. 2020) and 137 days, corresponding to the time a number of cured meat products were observed negative (Petrini et al. 2019), and finally a year.

### Single gene analysis

We ran a further analysis on three single genes that we extracted from the alignment, and we assessed the similarity of those across the alignment. We retrieved the B602L (1593 bp), I73R (219 bp), and MGF 360-10 L (1038 bp) gene sequences from Georgia 2007/1 reference sequence on GenBank [FR682468.2 (Chapman et al. 2011)]. All three genes are considered among the genetic or subgenotype markers (Gallardo et al. 2023, Mazloum et al. 2023).

The major capsid protein p72 (encoded by the gene B646L) is commonly used to define the virus genotype, while p54 [encoded by E183L gene and involved in the viral entry (Jia et al. 2017)] is also used to identify the genetic group (Gallardo et al. 2009). However, the gene B602L, located in the virus genome central variable region, can be used to discriminate between strains that are identical according to their p72 and p54 genotypes (Gallardo et al. 2009). I73R was found to be critical for ASFV pathogenesis as it suppresses the host innate response (Liu et al. 2023) and, finally, MGF360-10 L promotes the survival in host cells and might be responsible for immune response inhibition (Burrage et al. 2004, Li et al. 2023). We used the BLAST algorithm (Altschul et al. 1990) to extract the corresponding genome sections from the alignment, and we built a phylogenetic tree for each gene using *IQ-TREE2* (Minh et al. 2020).

**Figure 2:**
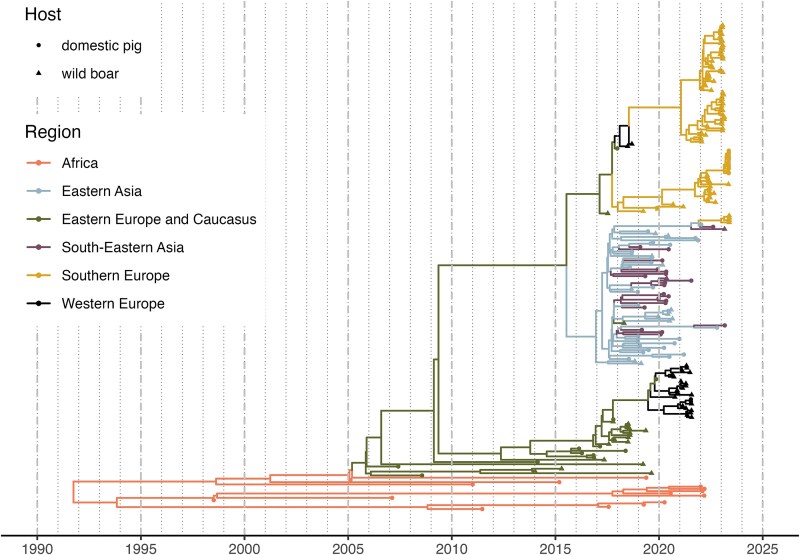
The maximum clade credibility (MCC) tree of the 217 ASFV whole-genome sequences used in the study. Branches are coloured according to the most likely region, while internal nodes and tips shape are determined by the observed (tips) or inferred (internal nodes) host (dots for domestic pigs or by triangles for wild boar or feral pigs).

## Results

### Time-scaled trees and global spread

After a preliminary sequence selection phase (Supplementary material, and Table S2), the final alignment we used for the analyses included 217 WGSs. The substitution model that best performed in our selection process was the Hasegawa–Kishino–Yano (HKY) (Hasegawa et al. 1985) with a Gamma-distributed heterogeneity model (Yang 1994), coupled with an exponentially distributed relaxed clock rate (Drummond et al. 2006) and the Bayesian SkyGrid (Gill et al. 2013) tree prior. The average clock rate estimate was 1.07 × 10^−5^ substitutions/site/year [95th high-posterior density, HPD, 0.82 × 10^−5^-1.36 × 10^−5^], which correspond to 1.97 substitutions/genome/year [HPD 1.51–2.51]. We did not observe any statistically significant variation in the clock rate estimates across region or host (Fig. S2). The estimated effective population size is reported in Fig. S3.

In Fig. 2, we reported the MCC tree with the addition of traits and branches coloured by region (see Figs S4 and S5 panel A for the branches coloured according to, respectively, the branches’ posterior probability estimate and host). According to the model estimates, the most recent common ancestor (MRCA) was in October 1993 [HPD February 1984—November 1998]. The MRCA of the Eurasian epidemic median estimate was November 2005 [HPD September 2003—May 2007], while the MRCA for the East Asian clade was December 2016 [HPD November 2015—September 2017].

**Figure 3:**
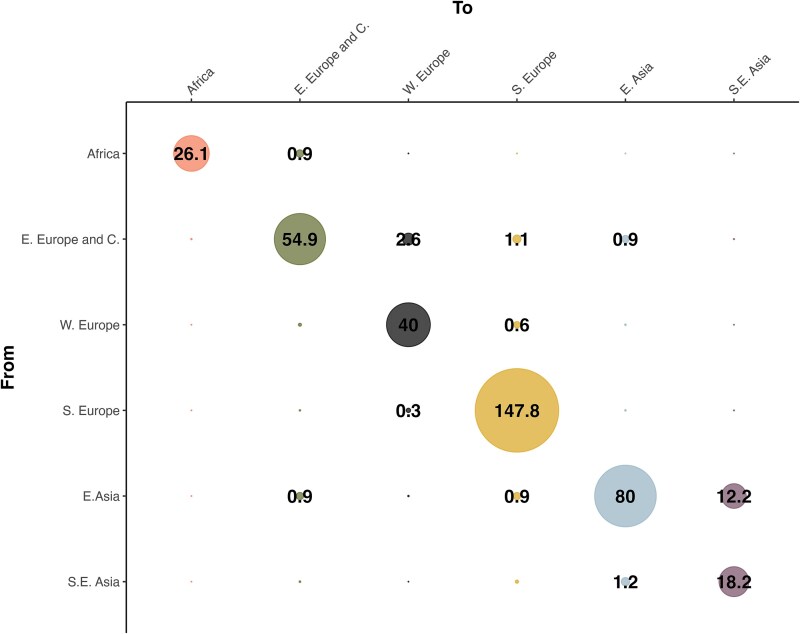
The estimated number of ASFV transitions within and between the six regions, across the 9001 sampled phylogenetic trees. The within-region number of transitions is positively correlated with the number of whole-genome sequences available for the region (Spearman ρ = 0.94).

**Figure 4:**
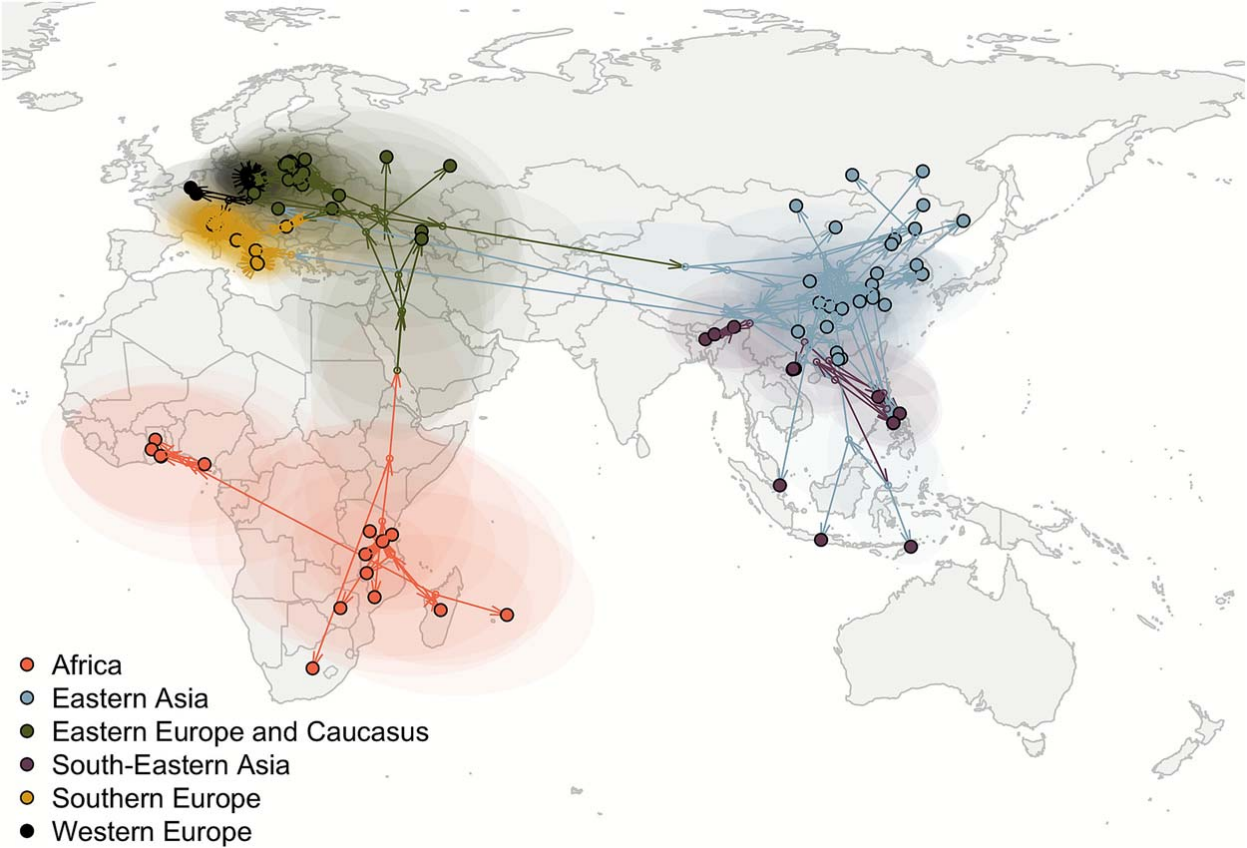
The spatially explicit MCC tree showing the global spread of ASFV estimated by *BEAST*. Black-circled dots represent sequences’ (i.e. tips) location (reported or centred within the smallest available administrative area), small dots represent the estimated internal nodes, arrows represent the tree’s branches, and transparent coloured areas correspond to the 80% high-posterior density. Colours correspond to the estimated (for branches or internal nodes) or observed (for tips) region.

The transmission matrix (Fig. 3) shows the number of estimated ASFV transitions within and between regions. The estimated number of within-region transitions is highly correlated to the number of sequences from each region (Spearman ρ = 0.94). As expected, the model estimated one transition from Africa to Eastern Europe and the Caucasus. Most of the other between-region transitions consist of single events (estimated transition value: ~1), with two exceptions: first, from Eastern to Western Europe (2.6). This may be a result of the introductions into Eastern Germany from the contiguous areas of Western Poland, which can be considered one cross-border cluster, with the strains also showing a characteristic tandem repeat in the O174L gene (Forth et al. 2023). The highest number of between-region transitions was observed from Eastern to South-Eastern Asia (12.2), which is of a similar magnitude to the South-Eastern Asia within-region transitions (18.2): this might have been facilitated by the trading links within countries of the area. Figure S5 reports the transition matrix between hosts (panel B): while between-host transitions are not negligible, our results show a dominant circulation within each host. The insight we can gather from this might be limited, however, because the internal nodes assignment of this trait could be easily biased by the available observations. By adding the coordinates as a continuous trait, *BEAST* estimated the global spatio-temporal spread of ASFV, which is reported in Fig. 4 (and a dynamic movie in Movie S1).

**Figure 5:**
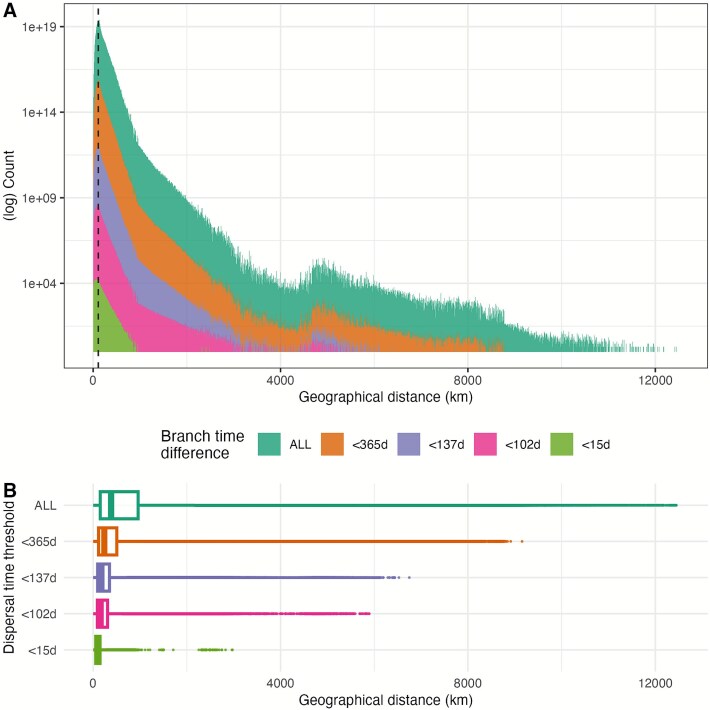
Distribution of the dispersal distance along branches, across all 9001 phylogenetic trees obtained in *BEAST*. The trees have been simplified by removing the nodes not located in areas where ASFV has been reported, and distances calculated between the remaining parent–child nodes (or tips). (A) Cumulative distribution of branches below a certain dispersal time threshold (from top to bottom: all dispersal estimates; below 365 days; below 137 days; below 102 days; below 15 days) and mode (110 km, dashed line). (B) Boxplot reporting the median and 25th/75th quantile of the dispersal distance values at different dispersal time thresholds.

After simplifying the tree as described in the methods section, the median [95th HPD] dispersal distance along the trees’ branches was 383.3 km [33.2–5276.3], while the mode was 110 km. If we consider the branches with duration less than a year (68.5% of the total), the median [95th HPD] distance was 158.2[19.7–1249.6] km. For branches with duration up to 137, 102, and 15 days (46.7%, 40.6%, and 11.2% of the total), the distances were, respectively, 146.1[18.8–941.9], 140.8[18.3–867.9], and 94.6[12.7–341.8] km. The cumulative dispersal distance distribution is reported in Fig. 5.

### The clusters in Italy

**Figure 6:**
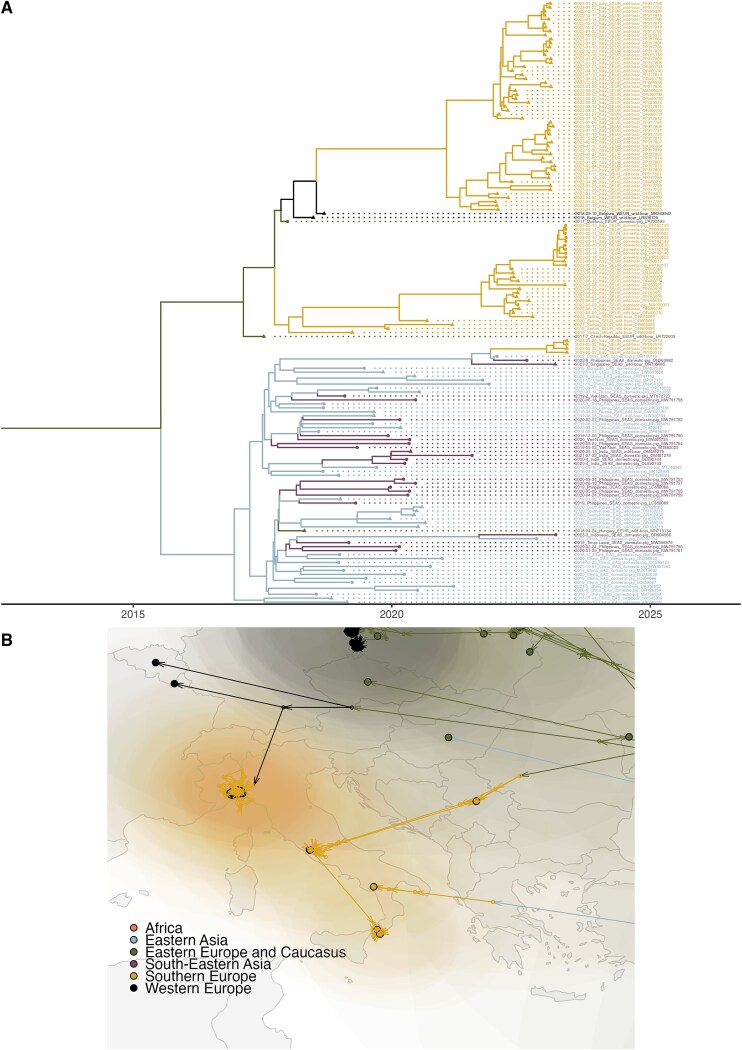
(A) A snapshot of the time-scaled ASFV MCC tree estimated by *BEAST*, showing the two clades with the presence of WGSs sampled in Italy (European clade, top, and wider East Asia clade, bottom). (B) The spatially explicit MCC tree showing the spread of ASFV focused on Italy.

The estimated median MRCAs of the four Italian clusters were January 2021 [HPD February 2020–September 2021] for the North-West, July 2021 [HPD August 2020–March 2022] for Rome municipality, January 2023 [HPD November 2022–March 2023] for Calabria, and February 2023 [HPD November 2022–May 2023] for Campania. Only two of the clusters were genetically similar, as in the MCC tree, the Calabria clade branches off the Rome one, which, in turn, is associated with sequences sampled in Serbia. Conversely, the North-West cluster sequences form a clade on their own, and the sequences in the same clade are two from Belgium and one from Moldova. Finally, the Campania cluster is genetically distinct, as these strains showed similarities with the Eastern Asian clade (Fig. 6).

**Figure 7:**
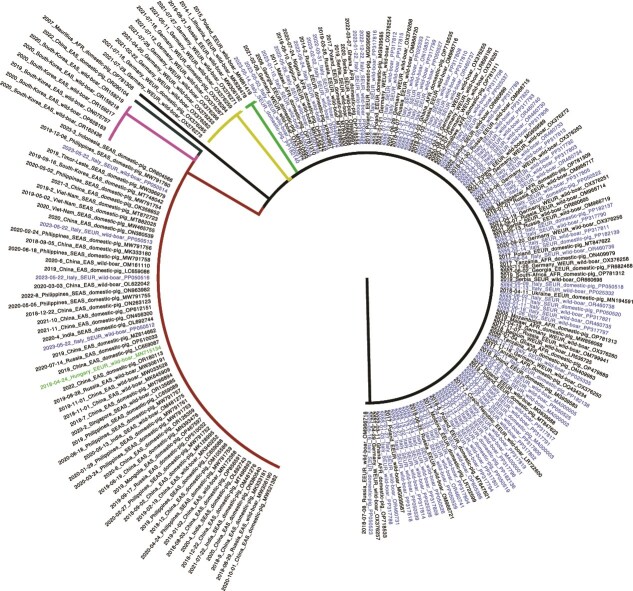
The phylogenetic tree of the ASFV MGF360-10 L gene. Branch colours represent clade: main clade, black; Eastern Europe clade (*n* = 3, 1 SNP), green; German clade, gold (*n* = 9, 1 SNP); wide Eastern Asia clade, red (*n* = 67, 1 SNP), South Korea clade, pink (*n* = 6, 2 SNPs). Single divergent sequences are coloured in black (China and Mauritius, both 2 SNPs). Italian sequences’ labels are coloured in blue and the Hungarian sequence in green (figure created with *FigTree v1.4.4*).

To confirm this result, we assessed the similarity across three individual genes extracted from the alignment. While the B602L and I73 genes were mostly conserved, in the gene MGF 360-10 L, one single-nucleotide polymorphism (SNP, position 986, A-to-G) was common to 67 sequences: 62 sampled in Eastern and South-Eastern Asia, 1 sequence from Hungary (MN715134), and 4 sequences from Campania, Italy (PP050512, PP050513, PP050514, and PP050516) (Fig. 7). Within this clade, six sequences from South Korea (ON075797, OR159217, OR159218, OR159219, OR162436, and OR281183) and one from China (OR2910104) had another SNP. Other separate and independent clades had one SNP (*n* = 9 from Germany, *n* = 3 from Russia, MW306192, Lithuania, MK628478, and Poland, MH681419), while one sequence from Mauritius (OP781308) had two SNPs, not observed in other sequences.

## Discussion

Phylodynamic approaches have been highly successful in describing the dynamics of viral infectious disease across spatial scales in human and animal populations (Lemey et al. 2014, Duchatel et al. 2019, Guinat et al. 2021). Here, we adopted this approach to enhance our understanding of the global spread of ASFV, focusing on genotype II. Our findings suggest that the current ASFV panzootic is the result of long-distance anthropogenic transmissions coupled with local spread caused by either wildlife or human activities in areas where the virus encroached. A similar result was obtained by Gámbaro and coauthors (Gámbaro et al. 2025), using a phylogeographic approach, they showed how the slow ASFV dispersal dynamic was coupled with long-distance ‘jumps’. Our results were further confirmed by calculating the association index and parsimony score (Parker et al. 2008) on the region trait. These scores were highly correlated with the phylogeny (Fig. S6), which is interpreted to be a sign of an intense within-region circulation, and a lower number of between-region transmissions (Parker et al. 2008), confirming the result reported in the transmission matrix (Fig. 3).

The maximum clade credibility tree allowed us to pinpoint several long-distance transmission events that characterized the global spread. While the first out-of-Africa movement of genotype II, which started this panzootic, and the movement from Europe to China were already well documented (Gaudreault et al. 2020, Njau et al. 2021, Rowlands et al. 2008), our model provided insights into other potential spreading events at the continental or intercontinental scale.

Italy is among the recently hit countries, where four clusters have been detected on the mainland since January 2022. Our analyses showed that at least three of these could likely be the results of independent introductions into the country. The clusters in the North-West and in the Rome municipality were caused by distinct ASFV strains, and their most recent common ancestor dates to long before the first Italian case was observed; therefore, a common origin within the country is unlikely. Previous studies employing a multi-gene approach reported similar conclusions, with the clusters in the North-West and Rome belonging to different genetic groups (Giammarioli et al. 2024). The sequences sampled in the Rome municipality showed similarities with those from Serbia, suggesting a potential epidemiological link between the two areas: these results are also consistent with another study (Gámbaro et al. 2025). Conversely, the North-Western cluster isolates grouped in the same clade as one sequence from Moldova and two from Belgium. The last observed case of the Belgium outbreak was reported in mid-2019 (Dellicour et al. 2020: 202); therefore, a direct link between the two clusters can be excluded. Conversely, because of the absence of recent WGSs from Moldova, direct links cannot be ruled out. The MCC tree showed the Calabrian clade closely associated with the Rome one, although the sequences in Calabria show distinctive deletions in their genome (Torresi et al. 2025). These deletions could have emerged in the area that was the source of both clusters or alternatively in Calabria. From the phylogeny, it seems less likely that the deletions emerged in the Rome cluster since it was resolved and none of these deletions were observed in the sampled sequences from Rome. Further investigations to test these hypotheses would require additional virus isolates to fill the knowledge gap.

Surprisingly, we found the Campanian sequences associated to the wider Eastern Asian clade at the whole genome level than the other Italian clades, and this was also confirmed using the single gene MGF 360-10 L. Specifically, we found the same SNP substitution in all East Asian sequences (both from East Asian and South-East Asia), in the Campania clade and in one sequence from Hungary, isolated in 2018. One potential explanation is that the substitution emerged in Europe, and then, this variant strain was introduced to Asia and Hungary. Independent of where this strain emerged, the Campania cluster likely represents a further introduction in continental Italy. Zhang and coauthors (Zhang et al. 2023b) also found the Hungarian sequences branching off from the East Asian clade.

The Italian clusters need to be considered in the wider context of the ASFV circulation, specifically within the multiple virus emergence in Central, Western, and Southern Europe, where many cases/outbreaks are in areas without a neighbouring affected wild boar population. Our model estimated up to seven clusters likely caused by new introductions: other than the three in Italy, the Czech Republic (detected in 2017), Hungary (2018), Belgium (2018), and Western Poland (2019). The latter further expanded to Germany, where it was first detected in 2020 (Sauter-Louis et al. 2021). We are also aware of at least two further introductions not reported in our study, in Sweden (Chenais et al. 2024) and Baden-Wurttemberg (Germany, 2022, personal communication). This situation is mirrored in South-Eastern Asia, where the pathogen was introduced in several countries, including many islands, in the span of a few years. The circulation in this area is well represented in the transition matrix, which shows how the transitions between East and South-East Asia were similar to the within-region ones. Another notable long-distance movement of ASFV was from South-Eastern to Western Africa, where it emerged in Nigeria in 2020 (Adedeji et al. 2021, Ambagala et al. 2023) and Ghana in 2022 (Spinard et al. 2023), although according to our estimates, it might have been circulating in the region as early as 2017.

The current analysis presented many challenges related to the pathogen itself and to its epidemiology. While phylogenetic and phylogeographic approaches have been mostly used to tackle fast-mutating RNA viruses, such as avian flu (Lycett et al. 2020) and Porcine Reproductive and Respiratory Syndrome virus (Makau et al. 2022), recent developments showed these can be applied to slow-evolving pathogens, such as *Staphylococcus aureus* (Yebra et al. 2022) or *Mycobacterium bovis* (Crispell et al. 2019, Rossi et al. 2023). ASFV could be considered in the middle of these variation-wise, with a substitution rate of approximately two substitutions/genome/year. The high similarity of many sequences, however, could have caused a low posterior probability value in some branches of the MCC tree (Fig. S4): this was the case not only for smaller clusters, like the North-Western Italian clade, but also for the wider Eastern Asian clade. To avoid further biases in the MCC tree, discrete and continuous traits were added in a second step.

Following the simplification of the trees to remove internal nodes in presumed ASFV-free regions, we analysed the resulting dispersal distance distribution across the 9001 trees. By considering only branches with dispersal time ≤137 days, which could potentially allow the virus survival in cured or refrigerated pork, the dispersal distance exceeded 900 km (Fig. 5), further highlighting the potential role of contaminated material movement in ASFV spread. If we consider dispersal times as short as 15 days, also comparable to the virus generation time, the median distance was ~95 km, and branches could be as long as 340 km, far beyond the potential reach of infected wildlife movements, suggesting the prominent human-mediated dispersal in this case as well. This analysis’ objective was to provide insights on the potential dispersal distance within time periods compatible with different transmission mechanisms; one limitation was that the current algorithm does not re-estimate the position of internal nodes in ‘credible areas’ (i.e. areas known to be affected by ASFV or adjacent), but it simply connects a tip (or an internal node) with the closest ancestor located in a positive ASFV area. Potential developments in this methodology could lead to more accurate estimates of internal node locations; however, this still presupposes that the reporting from truly ASFV-affected or free countries is accurate.

Similar to two previous studies (Shen et al. 2022, Zhang et al. 2023b), we estimated a clock rate in the order of 1–1.5 × 10^−5^ (1.07 × 10^−5^, compared to 1.14 × 10^−5^ and 1.31 × 10^−5^) but higher than another recent study (Gámbaro et al. 2025) (0.57 × 10^−5^). We employed an exponential relaxed clock model, whereas the first two studies used a strict model and the latter a logistic distributed clock. These results are reflected in the Eurasian epidemic MRCA estimate: our result (November 2005 [HPD September 2003–May 2007]) was similar to Zhang and co-authors (Zhang et al. 2023b) but with a narrower HPD interval (August 2005 [HPD April 2000–January 2007) but wider than Gámbaro and co-authors (Gámbaro et al. 2025) (HPD August 2004–September 2007).

A limitation is the notable absence of WGSs from Eastern Europe between 2008 and 2014 and the relatively small number of sequences from countries infected earlier in the epidemic. Isolates from these areas, and eventually time periods, could provide further insights on the early spread of the disease and therefore improve the robustness of the MCC tree estimates. Another important question is whether the introduction of ASFV into a naïve population is leading to the emergence of new strains or variants (Forth et al. 2023), in particular in the context where ticks are not involved in the spread and maintenance of the disease.

While our findings provide further insights on ASFV genotype II global circulation, a potential extension of this work could help to uncover social, economic, or environmental drivers—beyond the presence of wild boars—that enable its introduction and establishment in new regions. Determining which of these factors are associated with long-distance transmissions could be crucial to improve the biosecurity and surveillance in those areas deemed at higher risk.

## Conclusions

In conclusion, our phylogenetic model of ASFV genotype II reveals a complex dynamic including importations layered over local transmissions in both wild and domestic swine. The uncovering of multiple introductions into Europe—including at least three into Italy—and the potential genetic links to East Asian lineages expose critical blind spots. To bridge these gaps, genomic surveillance of the virus needs to be expanded and improved across under-sampled regions and to consider these data with ecological, trade, and socio-economic information, using complementary epidemiological approaches. Only through such an integrated, high-resolution monitoring framework can we sharpen real-time risk assessments, design context-specific biosecurity measures, and ultimately stop ASFV’s expansion.

## Supplementary Material

Rossi_et_al_ASFV_SupMat_veaf103

Rossi_et_al_ASFV_SM_Movie1_veaf103

## Data Availability

All the African Swine Fever virus genomes used in this study are deposited in NCBI GenBank (see Supplementary material for accession numbers).
