# Supplementary figures and images for: A phylogenetic contribution to understanding the panzootic spread of African swine fever: from the global to the local scale
Source: Virus Evol. 2025 Dec 24;12(1):veaf103. doi: 10.1093/ve/veaf103 (PMC12831188; doi:10.1093/ve/veaf103)

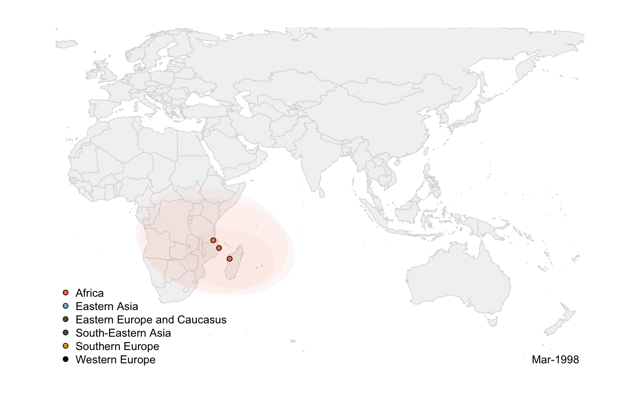

Supplement: Rossi_et_al_ASFV_SM_Movie1_veaf103 [file rossi_et_al_asfv_sm_movie1_veaf103.gif]
